# An explainable Bi-LSTM model for winter wheat yield prediction

**DOI:** 10.3389/fpls.2024.1491493

**Published:** 2025-01-17

**Authors:** Abhasha Joshi, Biswajeet Pradhan, Subrata Chakraborty, Renuganth Varatharajoo, Abdullah Alamri, Shilpa Gite, Chang-Wook Lee

**Affiliations:** ^1^ Centre for Advanced Modeling and Geospatial Information Systems (CAMGIS), School of Civil and Environmental Engineering, Faculty of Engineering & IT, University of Technology Sydney, Ultimo, NSW, Australia; ^2^ School of Science and Technology, Faculty of Science, Agriculture, Business and Law, University of New England, Armidale, NSW, Australia; ^3^ Department of Aerospace Engineering, University Putra Malaysia, UPM, Serdang, Malaysia; ^4^ Department of Geology and Geophysics, College of Science, King Saud University, Riyadh, Saudi Arabia; ^5^ Symbiosis Centre for Applied Artificial Intelligence, Symbiosis Institute of Technology, Symbiosis International Deemed University, Pune, India; ^6^ Department of Science Education, Kangwon National University, Chuncheon-si, Republic of Korea

**Keywords:** crop yield, explainability, shap, lime, integrated gradients, bidirectional LSTM

## Abstract

Accurate, reliable and transparent crop yield prediction is crucial for informed decision-making by governments, farmers, and businesses regarding food security as well as agricultural business and management. Deep learning (DL) methods, particularly Long Short-Term Memory networks, have emerged as one of the most widely used architectures in yield prediction studies, providing promising results. Although other sequential DL methods like 1D Convolutional Neural Networks (1D-CNN) and Bidirectional long short-term memory (Bi-LSTM) have shown high accuracy for various tasks, including crop yield prediction, their application in regional scale crop yield prediction remains largely unexplored. Interpretability is another pressing and challenging issue in DL-based crop yield prediction, a factor that ensures the reliability of the model. Thus, this study aims to develop and implement an explainable DL model capable of accurately predicting crop yield and providing explanations for the predictions. To achieve this, we developed three state-of-the-art sequential DL models: LSTM, 1D CNN, and Bi-LSTM. We then employed three popular interpretability techniques: Local interpretable model-agnostic explanations (LIME), Integrated Gradient (IG) and Shapley Additive Explanation (SHAP) to understand the decision-making process of the models. The Bi-LSTM model outperformed other models in terms of predictive performance (R^2^ up to 0.88) and generalizability across locations and ranges of yield data. Explainability analysis reveals that enhanced vegetation index (EVI), temperature and precipitation at later stages of crop growth are most important in determining Winter wheat yield. Further, we demonstrated that XAI methods can also be used to understand the decision-making process of the models, to understand instances such as high- and low-yield samples, to find possible explanations for erroneous predictions, and to identify regions impacted by particular stress. By employing advanced DL techniques along with an innovative approach to explainability, this study achieves highly accurate yield prediction while providing intuitive insights into the model’s decision-making process.

## 1 Introduction

Wheat is one of the three most widely consumed staple foods worldwide (FAO, 2022). Timely and reliable prediction of Wheat yield at a regional scale is a critical component of food security. Governments rely on crop yield predictions to ensure a stable food supply and to decide on import-export policies. Similarly, farmers, insurers and agribusinesses use this information for business and management decisions, including crop and insurance pricing, as well as supply chain management (Hoffman et al., 2015; Isengildina-Massa et al., 2008; Sherrick et al., 2014). As the global population grows and climate change becomes more severe, accurate and reliable crop yield prediction at a regional scale has become even more imperative. Advanced predictive models that combine high performance with interpretability can play a vital role in addressing these challenges.

There are mainly two approaches for crop yield prediction: process-based models (Corbeels et al., 2016; Keating et al., 2003), which simulate the physical processes of crop growth, and empirical data-driven models (Guerif et al., 1985; Kuwata and Shibasaki, 2016; Ma et al., 2021), which rely on historical data. Although processed-based models are transparent and can provide scientific explanations for their predictions, their calibration requires extensive local data and expertise. Environmental variability and the limited availability of spatially referenced input data can significantly affect the predictive accuracy of process-based models when used for larger areas (Folberth et al., 2016; Huang et al., 2015; Lobell et al., 2015).

An alternative approach to predicting crop yield is to use data-driven models (Guerif et al., 1985; Kushal and Khanal, 2023). These data-driven algorithms learn the relationship between input variables and crop yield without relying on explicit knowledge of crop science. Traditional statistical models are interpretable to a certain degree by assessing their coefficients but they are limited by their inability to model complex nonlinear relations between input features and the target variables. Due to the ability to account for the nonlinear relation, data availability, and better hardware, the use of advanced data-driven models like DL for large-scale crop yield has increased rapidly over the last few years (Jiang et al., 2020; Joshi et al., 2023b).

Long Short-Term Memory (LSTM) networks have emerged as one of the most widely used DL architectures in yield prediction studies providing promising results (Jiang et al., 2020; Joshi et al., 2023b; Muruganantham et al., 2022; Schwalbert et al., 2020; Wang et al., 2018). LSTM is a type of Recurrent Neural Network (RNN) that is designed to learn features from sequential data while addressing the vanishing gradient issue of traditional RNNs (Hochreiter and Schmidhuber, 1997). Besides LSTM, other networks designed to process sequential data include Bi-LSTM and 1D-CNN. 1D-CNN has demonstrated superior predictive performance compared to ridge regression and RF for wheat yield prediction in the Indian Wheat Belt (Wolanin et al., 2020), and has outperformed RF(rf), Support Vector Machine(SVM) and other traditional regression methods for winter wheat yield prediction in Germany (Srivastava et al., 2022). Meanwhile, a comparative analysis of LSTM, Bi-LSTM, and traditional models for rice crop detection revealed the superior performance of the Bi-LSTM architecture (Filho et al., 2020). Chen et al. (2022) showed that a Bi-LSTM model could effectively combine missing data imputation and crop classification, leading to improved classification performance. Recently, Saini et al. (2023) used BI-LSTM and CNN-based hybrid model to forecast sugarcane yield using historical yield and area data (without using remote sensing or environmental data) in India, outperforming LSTM, ARIMA, HoltWinter time-series, and Gaussian process regression methods. Although 1D-CNN and Bi-LSTM showed promising results for sequential data processing, including crop yield prediction, their application in regional-scale crop yield prediction is still limited compared to LSTM, and their performance against LSTMs remains largely unexplored.

Another crucial area of investigation lies in the interpretability of DL models used for crop yield prediction. Transparent models are generally considered more reliable, leading to higher confidence in decisions made based on their output. Without a clear understanding of how these models arrive at a certain yield value, there is often a lack of trust in their prediction. Explainability can also help identify the potential biases or errors in the model’s decision-making (Lapuschkin et al., 2019), and be used to refine the model architecture and feature selection (Matin and Pradhan, 2021). As previously mentioned, process-based and simpler statistical models are generally interpretable. Even some traditional machine learning models include built-in methods for assessing the relative importance of predictors on a global scale. However, DL models inherently lack transparency and are often described as “black box” models. These models contain thousands, if not millions, of weights across multiple interconnected nonlinear functions, making it challenging to interpret their decision-making processes. This poses a significant hurdle to their wider adoption in yield prediction.

The rapidly developing Explainable Artificial Intelligence (XAI) paradigm seeks to address this critical need for transparency and understanding within these black box models. In crop yield prediction, few studies have focused on explaining DL models. Wolanin et al. (2020) used regression activation maps in a 1D CNN architecture to visualize and analyze the model’s learned features and yield drivers. Srivastava et al. (2022) used SHAP to interpret their 1D-CNN model’s predictions and showcase explainability for feature selection. The study focused on meteorological and soil data and missed remote sensing data which is known to significantly influence yield. Recently (Mateo-Sanchis et al., 2023) used IG and SHAP to interpret the LSTM-based yield prediction model on a global scope. While the study leveraged two attribution methods with different theoretical foundations, its interpretability analysis primarily focused on identifying important features within the overall model potentially missing other insights. Thus, application of XAI in crop yield prediction is in its early stages.

In this context, the main aim to investigate the performance of different DL architecture while exploring interpretability for crop yield prediction at a regional scale. The main contributions of this work are described as follows:

- The study developed three deep learning designed to process sequential data (LSTM, 1D-CNN, and Bi-LSTM) along with a random forest (RF) model, a widely used traditional machine learning method, to predict crop yield using multitemporal remote sensing and weather data. To the best of our knowledge, this is the first study to employ a Bi-LSTM model for crop yield prediction using Earth observation data and to benchmark its performance against other state-of-the-art machine learning models.
- The study then employed three popular model-agnostic interpretability techniques, namely LIME, Integrated Gradients (IG), and SHAP, to understand the decision-making process of each DL model. The study demonstrated a novel way of using XAI to identify global feature importance in yield prediction, understand specific instances like high- and low-yield samples, provide potential explanations for incorrect predictions and identify regions impacted by specific stresses. This provided intuitive insights into the model’s decision-making process.

The rest of this paper is organized as follows: Section 2 depicts the proposed methodology, including the data used, the development, and the implementation of machine learning and interpretability methods. Section 3 presents the results achieved by our models. Section 4 discusses and provides comments on their significance. Finally, section 5 presents the conclusions of the paper.

## 2 Materials and methods

### 2.1 Study area

The study area comprised 606 winter wheat-growing counties in ten neighboring states in the U.S.: Kansas, Washington, Oklahoma, Colorado, Montana, Texas, Idaho, Nebraska, Oregon, and South Dakota. These ten states are among the top 11 producers of winter wheat in the USA and collectively contributed to approximately 70% of winter wheat production in the country over the last decade (USDA, 2023). Notably, the USA is the world’s fourth-largest producer and second-largest exporter of Wheat (FAO, 2021; USDA, 2021), and around 70-80% of the country’s wheat production comes from winter wheat varieties (USDA-NASS, 2021). The study area comprises a diverse range of climates, including arid, temperate, and cold conditions (Beck et al., 2018).

### 2.2 Dataset and pre-processing

The study used various publicly available data spanning the entire winter wheat growing season, from September to July, across the USA (2008-2021). Google Earth Engine (Gorelick et al., 2017) and ArcGIS were used for various data pre-processing, including data selection, filtering, aggregating data to the county level, and converting data to the desired format. The preprocessing steps were performed to obtain noise-free county-level data suitable for use as input to the model. First, only cloud-free remote sensing data were selected, and fortnightly remote sensing observations were aggregated to monthly intervals to match the monthly frequency of weather data, as described in Section 2.2.2. Then, all input variable values at the pixel level were averaged at the county level. Instances with missing values of one or more variable were then removed from the dataset. Lastly, prior to model training, all predicting variables were normalized to have a mean of 0 and a standard deviation of 1. The normalization process improves model convergence and prevents variables with larger magnitudes from dominating the learning process.

#### 2.2.1 Crop yield and crop mask data

**Figure 1:**
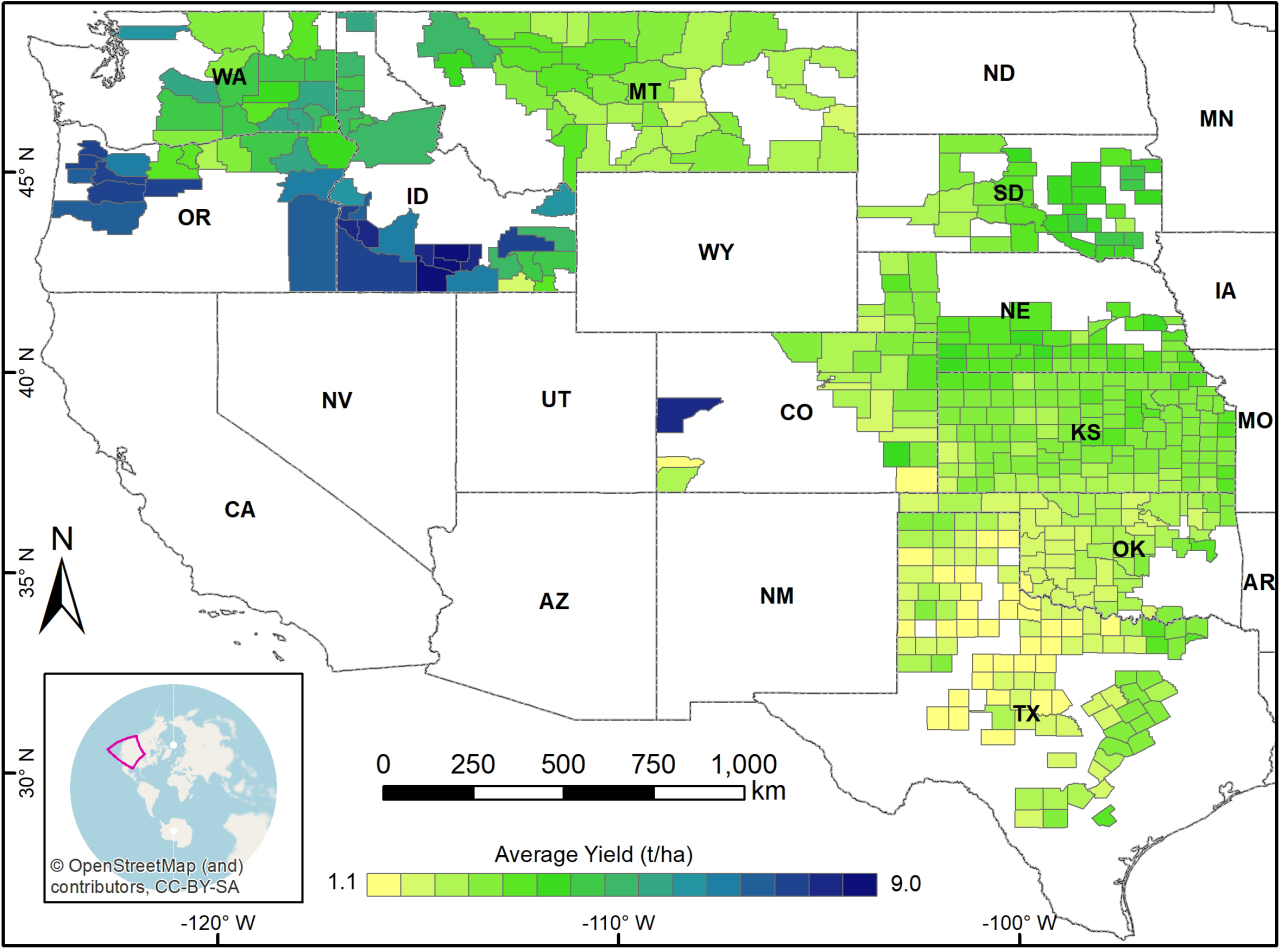
Study area showing the spatial distribution of average winter wheat yield across the counties from 2008 to 2021.

**Figure 2:**
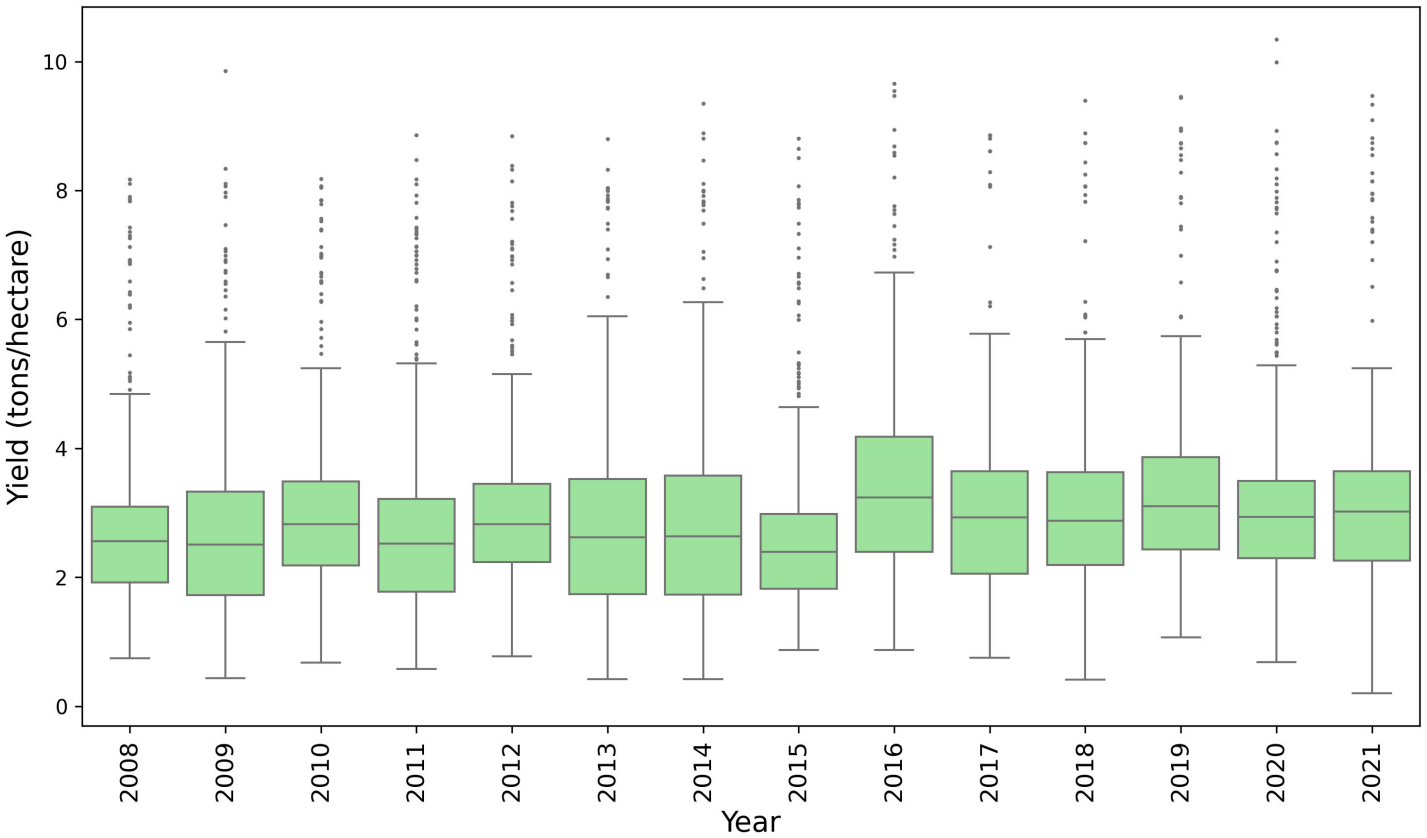
Boxplot of the county-level winter wheat yields from the study area for the year 2008 -2021.

County-level winter wheat yield records were obtained from the National Agricultural Statistics Service (NASS) database of the United States Department of Agriculture (USDA) (USDA, 2023). Data from 2008 to 2021 were used to train, validate, and test our model. **Figure 1** illustrates the spatial distribution of average winter wheat yield for each county from 2008 to 2021, and **Figure 2** shows the temporal distribution over the study period. To identify the winter wheat area within each county, we utilized the USDA-NASS cropland data layers (CDLs) (NASS, 2022). These annual, crop-specific maps are generated using moderate-resolution satellite imagery and ground-truth data.

#### 2.2.2 Satellite data

Satellite-derived vegetation indices such as the normalized difference vegetation index (NDVI) and the enhanced vegetation index (EVI) are commonly used for crop yield prediction. These vegetation indices are related to the photosynthetic activity of the plant and provide an important measure for monitoring plant growth, health, and productivity. This study employed the EVI derived from the MODIS Terra MOD13Q1 V6.1 product. EVI provides a quantitative measure of vegetation greenness while accounting for factors like soil reflectance and atmospheric haze, leading to a more reliable indicator of vegetation growth. While both EVI and NDVI are highly correlated, EVI was found to be more effective than NDVI in predicting wheat yield in the U.S (Joshi et al., 2023a). The MOD13Q1 V6.1 product is a 16-day spatiotemporal dataset providing high-resolution (250 m) vegetation indices and surface reflectance measurements. The data collected over 16-day intervals were transformed into monthly data through a weighted average method, with the weights determined by the extent of temporal overlap. Only pixels with high-quality data were considered for acquiring the time-series data. Pixels affected by snow or ice coverage, or obscured by clouds, were masked. EVI is calculated as shown in Equation 1.


(1) $$EVI=2.5\;\times \;\frac{\left(NIR\;-\;Red\right)}{NIR\;+\;C1\;\times \;Red\;-\;C2\;\times \;Blue\;+\;L}$$

where, Red and NIR are the atmospherically corrected surface reflectance in the red and near-infrared channels, the variables L correspond to the soil and canopy background adjustment factor, and C1 and C2 represent coefficients for correcting atmospheric influences. The specified value used in the MODIS product are L = 1, C1 = 6, and C2 = 7.5.

#### 2.2.3 Meteorological data

Meteorological data provides insight into essential environmental factors that affect crop growth and yield. Meteorological data is also commonly employed in various yield estimation methods, including process-based, statistical, and machine-learning approaches at regional or field scale. The meteorological data were extracted from the TerraClimate dataset (Abatzoglou et al., 2018), which provides global climate data at the monthly level at ~4-km (1/24th degree) spatial resolution. The specific meteorological variables used in this study are maximum temperature (tmmx), precipitation accumulation (pr), reference evapotranspiration (pet), and wind speed (vs). These variables were selected to represent key climate-related factors including temperature-related dynamics, water supply, water demand, and atmospheric conditions.

### 2.3 ML models

This study employed one widely used robust traditional ML method, RF, and three state-of-the-art DL-based models: 1D Convolutional Neural Network (1D CNN), Long Short-Term Memory (LSTM), and Bidirectional LSTM (Bi-LSTM) to predict winter wheat yields. These DL models have proven effective at capturing complex temporal dependencies within data. Mean squared error was used as the loss function in all the models. To find the best hyperparameter for each model, we used the grid search technique and the three-fold cross-validation approach (Bengio and Grandvalet, 2003; Kohavi, 1995). Data from the years 2008 to 2018 were used for hyperparameter tuning and training the models. The models were then used to predict the winter wheat yield for the year 2019, 2020, and 2021 to test the model. The models were implemented in the Python 3.10.6 environment. Scikit-learn 1.1.2 (Pedregosa et al., 2011) was used for the RF model, while TensorFlow 2.10 (Abadi et al., 2016) was used to build and train the LSTM, 1D CNN, and Bi-LSTM models. The models were trained on a high-performance computing (HPC) server equipped with Intel Xeon Gold 6238R processor running at 2.2GHz with 28 cores, 180GB RAM (Six Channel) and a NVIDIA Quadro RTX 6000 Passive GPU, with 4608 cores, 576 Tensor Cores, and 24GB of memory.

The hyperparameter tuning process for the Bi-LSTM model took 9235 seconds, LSTM took 5402 seconds, and 1D CNN took 3672 seconds. A total of 405 models were trained during this phase. The RF model required significantly less time, training 324 models only in 284 seconds. Once the optimal hyperparameters were determined, training the DL models took between 26 and 59 seconds, while the RF model required 16 seconds on the above-mentioned server.

#### 2.3.1 RF

RF is an ensemble learning method that uses multiple decision trees to reach a decision in a classification or a regression task (Breiman, 2001). Different decision trees are built and trained using a random subset of the training data and a random subset of features for each split, and averaging is used to improve the predictive performance. RF is widely used for predicting crop yield and has demonstrated superior performance compared to linear regression, decision trees, and even Scalable Vector Machine (SVM) (Khanal et al., 2018; Ramos et al., 2020). RF has also been found to be better at handling high-dimensional data (Ma et al., 2020). We used RF as a representative of the traditional ML approach. The specific values of hyperparameters used for RF are as follows: the number of trees was set to 100, the maximum depth of the tree was set to 20, the minimum number of samples required to be at a leaf node was set to 4, the minimum number of samples required to split an internal node was 10, and the number of features to consider when looking for the best split is the square root of the number of features.

#### 2.3.2 1D-CNN model

Convolutional neural networks (CNNs) are specialized artificial neural networks designed to process data with grid-like structures (Goodfellow et al., 2016). While 2D CNNs are more common for handling 2D data like images, 1D CNNs are particularly well-suited for sequential data, such as time-series. The unique feature of 1D CNNs is their significantly lower computational complexity. We constructed a model with three 1D convolutional layers, two 1D max-pooling layers, and two fully connected layers. Although dropout layers are more commonly used after dense layers, our experiment showed that incorporating dropout in our 1D CNN led to improved generalization, so we included the dropout layer after the convolutional layer as well. We utilized the Stochastic Gradient Descent (SGD) optimizer with a learning rate of 0.01 a batch size of 50, and trained the model for 50 epochs as determined through tuning.

#### 2.3.3 Long short-term memory

LSTM is an improved type of recurrent neural network (RNN), which addresses the vanishing and exploding gradient problem of vanilla RNNs and was originally proposed in 1997 (Hochreiter and Schmidhuber, 1997). LSTM cells can capture and remember information in very long sequences, controlled by three component input gates, forget gates, and output gates. The proposed architecture consists of two LSTM layers for capturing temporal patterns, a dropout layer for regularization, and a dense layer for yield prediction. The specific hyperparameters adopted are as follows: learning rate set to 0.0001, optimizer set to Adam, number of epochs set to 200, and batch size set to 50.

#### 2.3.4 Bidirectional long short-term memory

**Figure 3:**
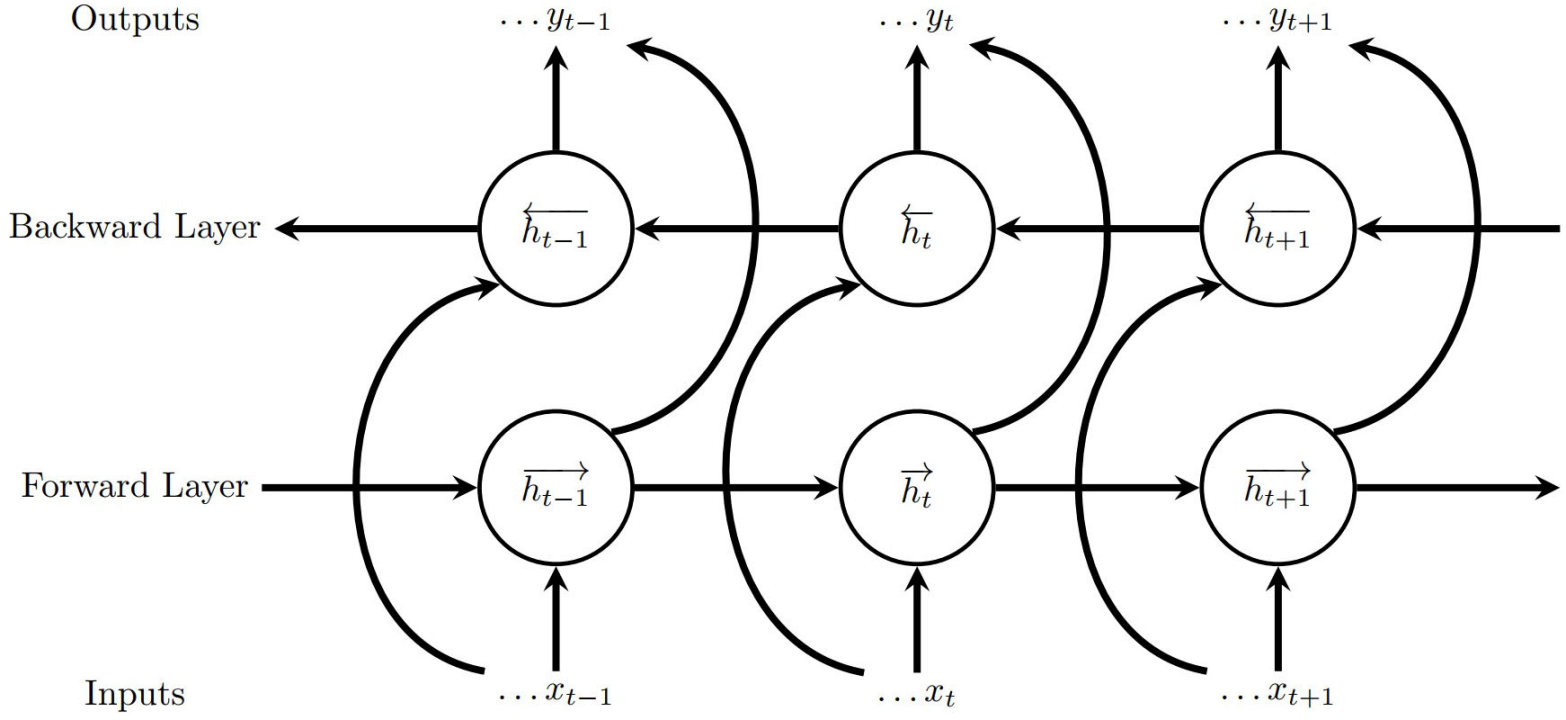
Example of Bi-LSTM Architecture.

**Figure 4:**
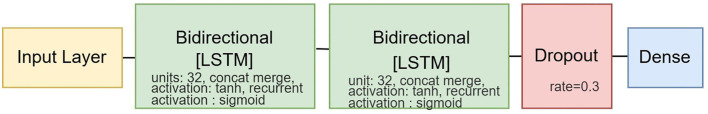
Bi-LSTM model implemented in the study.

A Bi-LSTM model consists of two LSTM components that process the data in both forward and backward directions simultaneously (Graves and Schmidhuber, 2005; Schuster and Paliwal, 1997). By processing the input data in both forward and backward directions, this method enables more comprehensive and robust learning of sequential features. The architecture of the Bi-LSTM unit consists of two LSTM components, one processing the input sequence as-is and the other in reverse, the output of which is the combined output of both components (**Figure 3**). Our model (**Figure 4**) consists of two bidirectional LSTM layers followed by a dropout for regularization after the second bidirectional LSTM layer, and a dense layer for predicting yield. The optimal hyperparameters chosen for this model via grid search are Stochastic Gradient Descent optimizer, a learning rate of 0.01 for 200 epochs, and a batch size of 50.

### 2.4 Predictive performance

To assess the accuracy and goodness-of-fit of our predictive model, we compared its predictions with actual data using two established metrics: mean-absolute-error (MAE) and coefficient of determination (R²). MAE quantifies the magnitude of prediction discrepancies (lower is better) and is in the same unit as the yield data. R² measures the proportion of variance in the actual data that is explained by the model. It reveals the strength of the model’s fit to the data where higher (closer to 1) signifies a stronger fit. The formula is as follows:


(2) $$R^{2}=1-\frac{\sum_{i=1}^{n}{\left(Y_{i}-\hat{Y_{i}}\right)}^{2}}{\sum_{i=1}^{n}{\left(Y_{i}-\bar{Y}\right)}^{2}}$$

(3) $$MAE=\frac{1}{n}\sum_{i=1}^{n}\left|Y_{i}-\hat{Y_{i}}\right|$$

where, $Y_{i}$ represents the actual yield values, $\hat{Y_{i}}$ represents the predicted yield values, and $\bar{Y}$ represents the mean of the actual yield values.

### 2.5 Explainability module

Explainable Artificial Intelligence (XAI) provides insight into the decision-making processes of ML models. XAI methods could be model-agnostic or model-specific. Model agnostic methods are designed to interpret any ML model irrespective of the model’s internal working. On the other hand, model-specific methods are tied to specific models and use the model’s intrinsic characteristics. Based on the scope of interpretability, XAI methods could provide local or global interpretability. This study employed three model-agnostic *post-hoc* XAI tools -SHAP, LIME, and Integrated Gradients- to explain model prediction on the local and global scope.

Local interpretable model-agnostic explanations (LIME) is a popular model agnostic method explainability approach developed in 2016 (Ribeiro et al., 2016). LIME tackles the challenge of black box models by constructing a simpler, interpretable model that approximates the prediction of the original model. The key lies in training this surrogate model on different interpretable features extracted from the original model’s inputs. LIME aims to provide locally faithful and interpretable approximation g of black box model f at a specific point z by solving the following optimization problem.


(4) $$g\left(z^{\prime }\right)=\arg\underset{g}{\min}L\left(f,g,\pi_{z}\right)+\Omega \left(g\right)$$

where, $z^{\prime }$ set of perturbed data points, $\pi_{z}$ is a proximity measure that gives higher weights to instances closer to the original instance, Ω(g) is a regularization term encouraging simplicity in the interpretable model), $\text{L}\left(f,g,\pi_{z}\right)$ is a loss function measuring the discrepancy between the predictions of g and f on the perturbed data points.

Gradient-based methods (Baehrens et al., 2010) are another family of popular techniques for interpreting ML models by attributing the contribution of each input feature to a given prediction. They work by calculating the gradient of the model output with respect to each input. The different methods include Saliency maps (Simonyan et al., 2013), DeconvNet, Guided Backpropagation. These methods rely on the assumption that predictions change linearly with respect to input features, leading to inaccurate interpretations when the model is highly nonlinear. (Sundararajan et al., 2017). To address this, (Sundararajan et al., 2017) designed a new attribution method called Integrated Gradients. Instead of simply looking at the gradient at the input, IG integrates the gradient’s influence along a path from a baseline to the actual input. This integration captures the cumulative contribution of each feature to the final prediction. The Integrated Gradients (IG) for a specific input feature $x_{i}$ can be mathematically defined as follows:


(5) $$IG_{i}\left(x\right)=\left(x_{i}-x_{i}^{\prime }\right)\times \int_{\alpha =0}^{1}\frac{\partial F\left(\alpha x+\left(1-\alpha \right)x^{\prime }\right)}{\partial x_{i}}\;d$$

$x_{i}$ is the value of the i^th^ feature in the actual input $x_{i}^{'}$ is the value of the i^th^ feature in the baseline input. F (x) is the output of the model for input x and $\frac{\partial F}{\partial x_{i}}$ is the partial derivative of the model’s output with respect to the i^th^ input feature.

The third framework used for explaining the DL models was SHAP, which is an implementation of Shapely values. SHAP (SHapley Additive exPlanation) is a recently developed *post-hoc* model agnostic approach framework that uses Shapley values to explain the output of ML models (Lundberg and Lee, 2017). Shapley originated from coalitional game theory and was originally designed to allocate credit among players in a multiplayer game (Shapley, 1953). It is calculated by averaging a player’s marginal contribution to all possible permutations of remaining players. For a specific instance x, the SHAP value $S_{i}\left(x\right)$ for feature i is defined as follows:


(6) $$S_{i}\left(x\right)=\phi_{i}\left(x\right)=\sum_{S\subseteq N\setminus \left\{i\right\}}\frac{\left|S\right|!\cdot \left(\left|N\right|-\left|S\right|-1\right)!}{\left|N\right|!}\left[f\left(x_{S\cup^{\;}\left\{i\right\}}\right)-f\left(x_{S}\right)\right]$$

where, N is set of all features, S is subset of features excluding feature i, |S| represents number of features in subset S, $f\left(x_{S}\right)\;$ is the model’s prediction when considering only the features in Subset S and $f\left(x_{S\cup^{\;}\left\{i\right\}}\right)$ is the model’s prediction when considering both features in subset S and feature i.

Shapley values are obtained by evaluating all possible combinations of features for each instance. This can be computationally complex, especially when there is a large number of features. So, SHAP turns the Shapley values method into an optimization problem and uses different SHAP explainers to approximate these values efficiently (Lundberg and Lee, 2017). In this study, we used Deep Explainer, an approach developed for a neural network-based architecture to approximate SHAP values for DL models. Deepexplainer is an improved version of DeepLIFT (Deep SHAP) that leverages background samples to approximate SHAP values. For the RF model, we employed Tree SHAP to compute the values.

SHAP python library was used to compute the SHAP value, and XPlique (Fel et al., 2022) tool was used to compute LIME and IG attribution values.

## 3 Result

### 3.1 Performance of different ML models

The study evaluated the performance of RF, 1D-CNN, LSTM, and Bi-LSTM models for winter wheat yield prediction across ten US states. Models were trained on data from 2008 to 2018 and used to predict yields for 2019, 2020, and 2021, testing their generalizability. Ten independent training runs were conducted for each model to account for training stochasticity, and the results were averaged. **Table 1** presents the evaluation metrics R² and MAE (t/ha) for each model across these three years.

**Table 1:** The R^2^ and MAE for the models in the year 2019, 2020 and 2021.

Year	R^2^				MAE			
	RF	1D-CNN	LSTM	Bi-LSTM	RF	1D-CNN	LSTM	Bi-LSTM
2019	0.82	0.76	0.80	0.84	0.49	0.58	0.54	0.48
2020	0.82	0.83	0.86	0.88	0.47	0.48	0.46	0.41
2021	0.65	0.73	0.78	0.78	0.68	0.63	0.56	0.56
Mean	0.76	0.77	0.81	0.83	0.55	0.56	0.51	0.48

Overall, all DL models demonstrated good predictive capabilities for winter wheat yield at the county level, with R² values ranging from 0.73 to 0.88. The Bi-LSTM model consistently displayed the highest R² and the least MAE among all the models, suggesting a slight advantage in explaining data variability. The LSTM followed the ranking, achieving an average R² of 0.81 from 2019 to 2021. The 1D- CNN’s R² ranged from 0.73 to 0.83 with an average of 0.77 for the three years. While RF also showed strong performance for 2019 and 2020, with R² values of 0.82 for both years, it showed a notable reduction in the R² value to 0.65 for 2021. This change in performance indicates that our RF model was unable to capture changes in the underlying patterns of the data well in 2021. Notably, the performance of all other models also declined in 2021, but the RF model generalized the least. MAE values follow a similar pattern to R² and range from 0.41 (for Bi-LSTM) to 0.68 (for RF).

**Figure 5:**
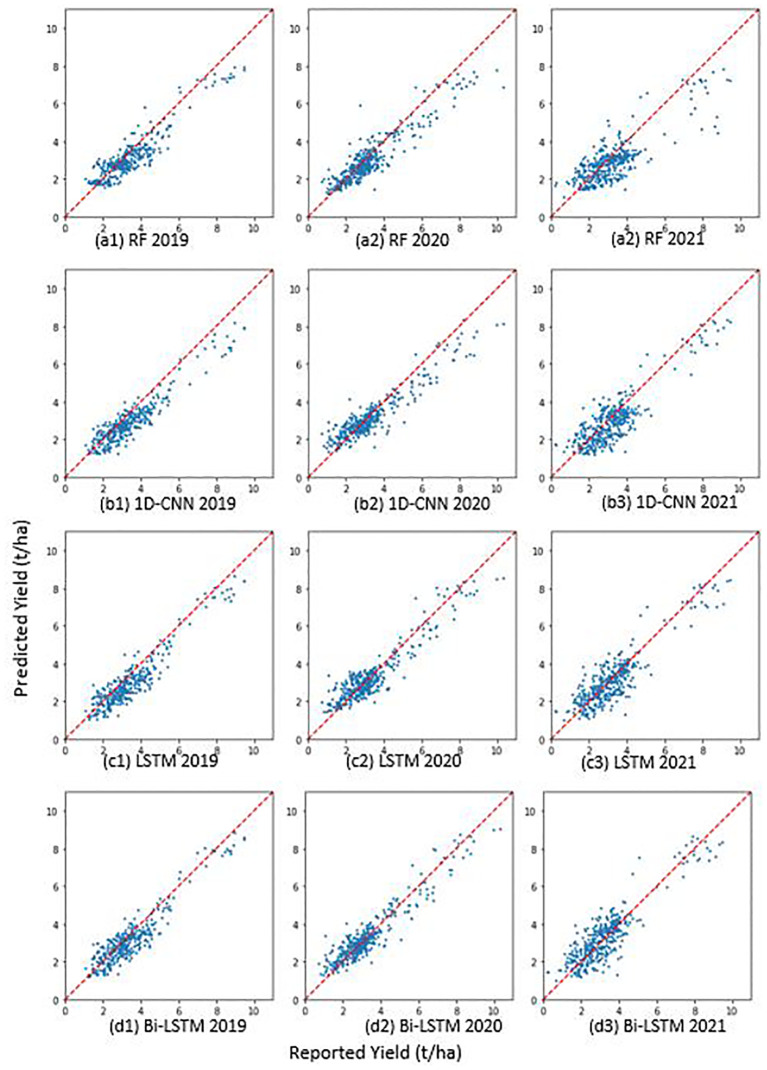
Plots depict reported vs. predicted yields for four models **(A–D)** in three testing years (1-3). (**A**: RF, **B**: 1D-CNN, **C**: LSTM, **D**: Bi-LSTM, 1: 2019, 2: 2020, 3: 2021).

The scatter plot (**Figure 5**) reconfirmed the strong agreement between the estimated and reported winter wheat yields at the county level in all models. Further, the graph shows a consistent pattern of under prediction for counties with higher yield values across all models. This is more evident in the 1D-CNN and RF model. It is important to note that the Bi-LSTM model was least affected by this phenomenon. One of the reasons for under prediction could be a smaller number of training samples with higher yield values.

**Figure 6:**
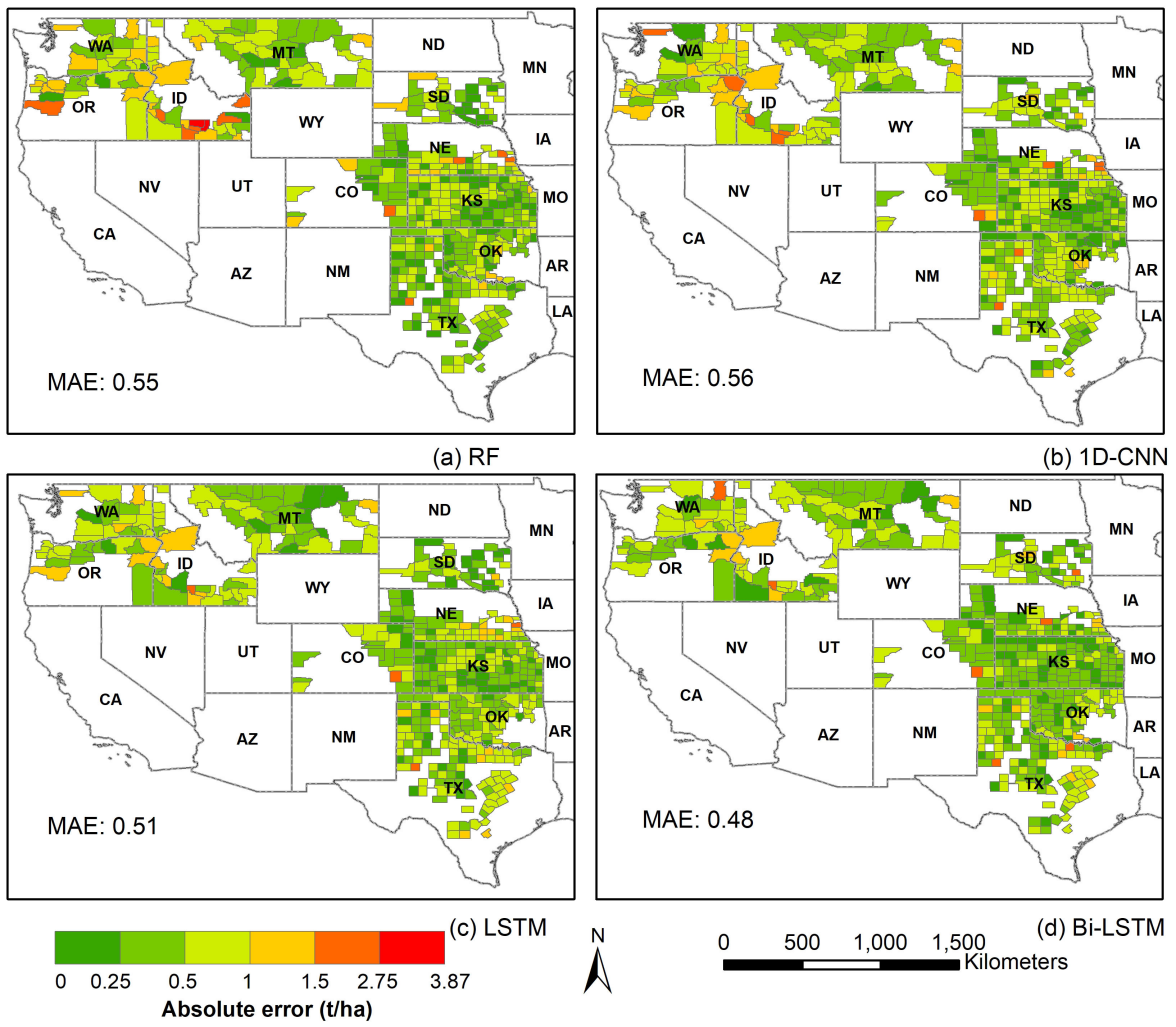
The absolute error maps of **(A)** RF, **(B)** 1D-CNN, **(C)** LSTM, and **(D)** Bi-LSTM model, averaged over the years 2019, 2020, and 2021. The mean absolute error of each model is also printed on the maps.

To further illustrate the performances of different models and assess the spatial generalizability of different models across various counties, we presented the maps of absolute error averaged for the years 2019, 2020 and 2021 (**Figure 6**). Absolute errors are generally higher for all models in the counties of Idaho, Washington, and Oregon. Notably, these regions also exhibit higher yield magnitudes (**Figure 1**). Comparing the models, the Bi-LSTM model demonstrated consistently smaller errors across most counties, and RF and 1D-CNN showed relatively higher errors. This indicates superior overall generalizability of the Bi-LSTM model. The mean absolute error (MAE) of all counties, averaged across the three years, is also the lowest for the BI-LSTM model.

### 3.2 Explaining the model

**Figure 7:**
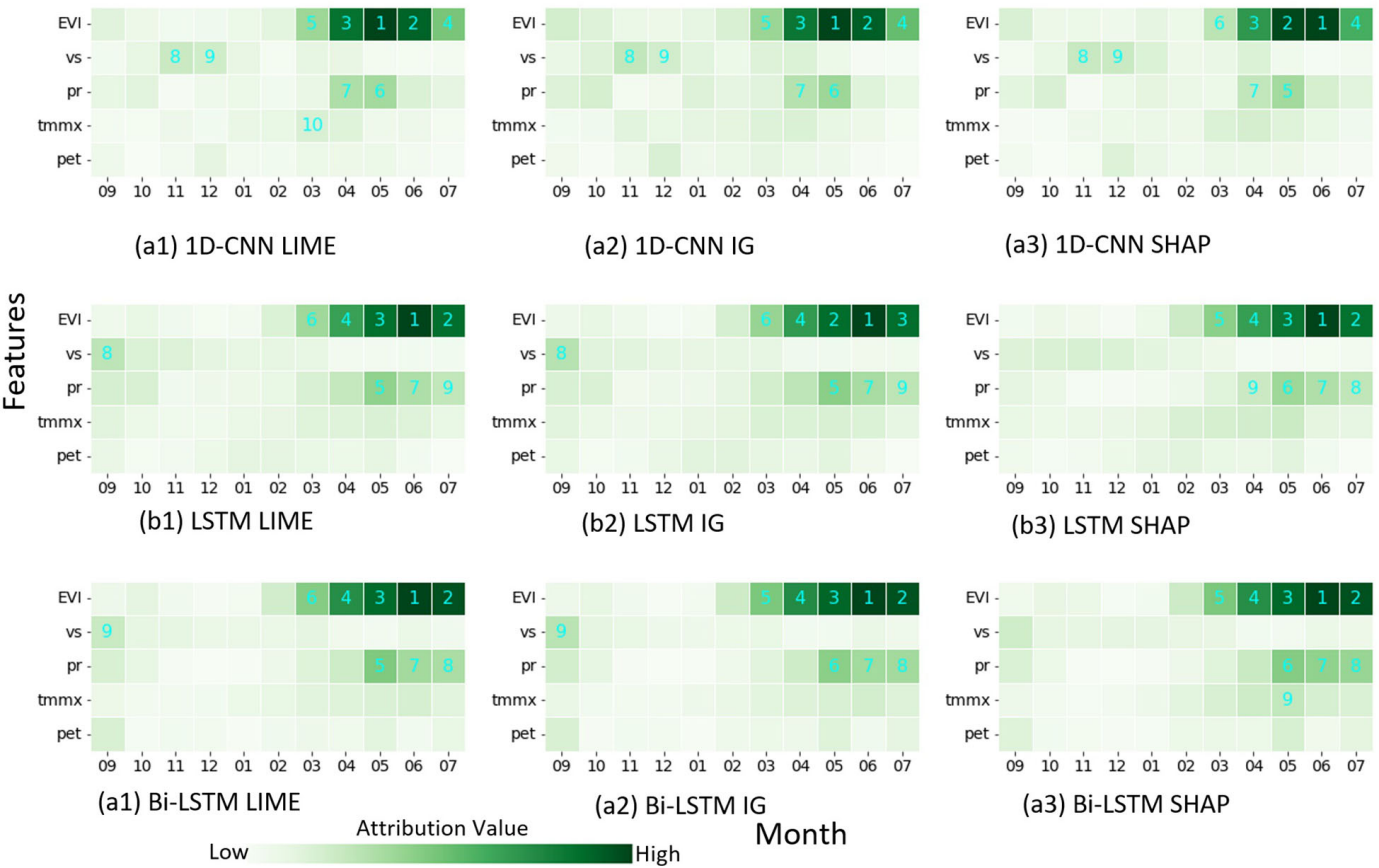
Heatmap showing global feature importance scores for three models (**(A)** 1D-CNN, **(B)** LSTM, and **(C)** Bi-LSTM) based on three interpretation methods ((1) LIME, (2) IG, and (3) SHAP). The number in the heatmap represents the importance ranking of the feature. The importance was derived by computing absolute mean of attribution values across all instances.

To investigate the decision-making process of the models, we employed various explainability techniques. First, we computed feature attributions using LIME, IG, and SHAP. We then derived the absolute mean of these values across all samples, which act as the global importance score provided by each attribution method. We visualized these feature importance scores as heatmaps, illustrating the mean impact of all data on the model output (**Figure 7**). Each row along the y-axis corresponds to a specific input feature, and each column along the x-axis corresponds to a different month. As we are interested in the relative contributions of different features rather than their specific attribution values, we excluded individual color bars and mapped the values to varying shades of green. Darker shades in the heatmap represent a higher contribution of the corresponding feature (as shown by the y-axis) for that month (as shown by the x-axis). We also ranked features based on their score. The top 9 features with the highest mean impact are identified by numbers superimposed on the heatmap.

Global explanations generated by averaging absolute feature attribution scores show a high degree of agreement, although not completely identical, in feature ranking across all three XAI methods: LIME, IG and SHAP. Furthermore, different DL models gave importance to similar features during decision-making, as shown by the similar heatmaps of different models. The explanation provided by the three attribution methods was consistent for individual predictions as well (not presented here). The heatmaps show that EVIs from March to pre-harvest are the most crucial factor determining yield across all models and explainability methods. Particularly, EVI in June, which is the time of physiological maturity, shows the highest predictive power and is ranked as either the most important or second most important feature in all plots. Precipitation accumulation between May and harvest follows EVI in terms of importance. Maximum temperatures during the same period also contribute, as evidenced by their relatively darker shades in most of the heatmap. Interestingly, wind speed in September also emerges as an important feature across various heatmaps.

**Figure 8:**
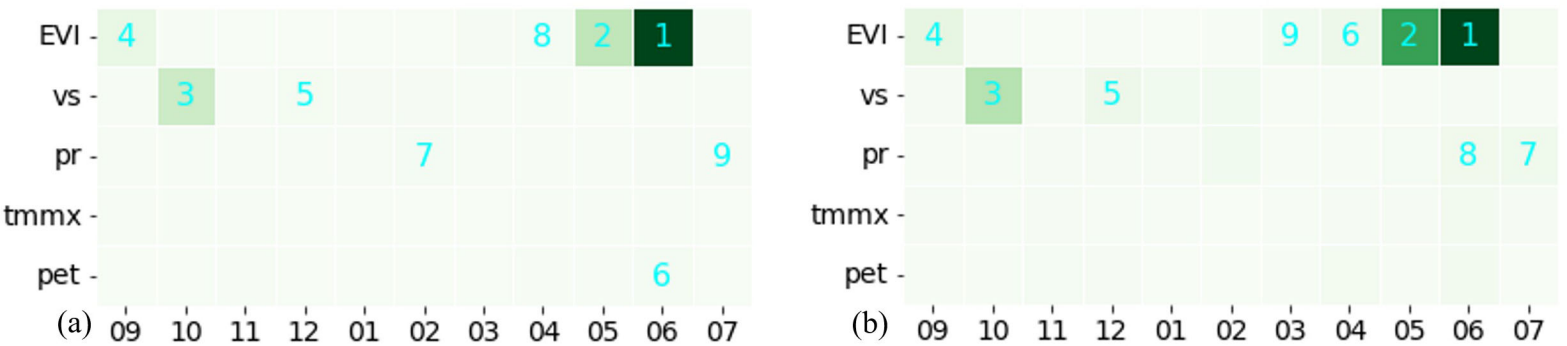
Attribution scores for RF model using: **(A)** Gini Importance, and **(B)** SHAP.

IG and SHAP Deep Explainer are designed for neural network models and cannot be applied to RF. To gain insights into the decision-making process of the RF model and compare it with the DL model, we employed TreeSHAP, a SHAP-based method specifically tailored for tree-based structures. Additionally, we examined the Gini importance of the RF model, a feature importance score generated by the RF model itself in scikitlearn. The Gini importance (Breiman, 2001) quantifies the average influence of each variable on reducing the impurity in the decision tree nodes. Our analysis revealed that EVI in June ranks as a highly important feature in both important measures (**Figure 8**). It is important to note that the model prioritizes a few key variables and assigns lower importance to others. The other features that stand out include EVI in May, June and September and windspeed in October.

#### 3.2.1 SHAP explanation of Bi-LSTM model

As the explanation provided by the three attribution methods were consistent, in the rest of the result section, we used only the SHAP method to explain the predictions made by our best-performing model: Bi-LSTM.

**Figure 9:**
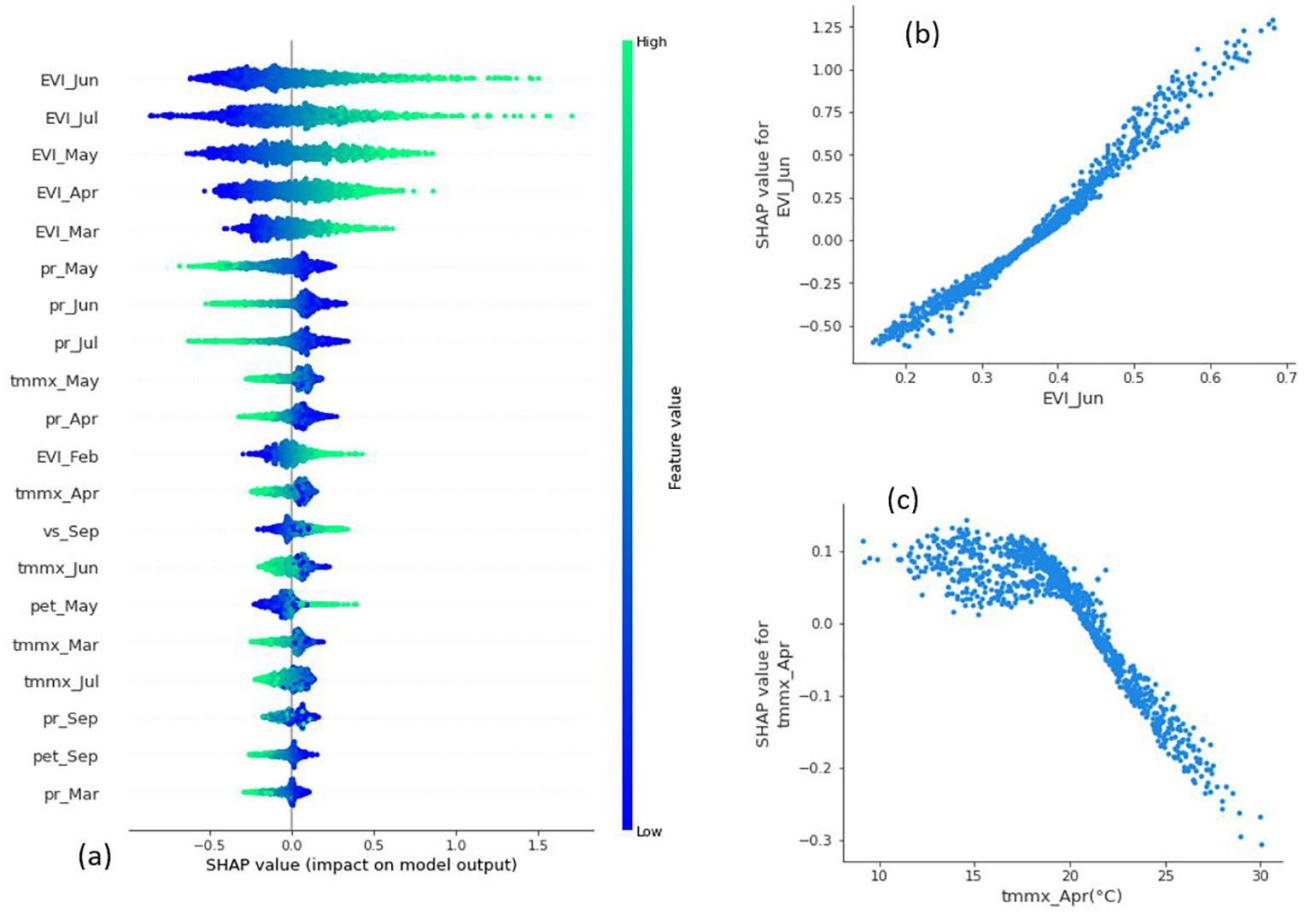
SHAP summary and dependence plots. **(A)** Summary plot showing the 20 most influential features in order of importance in the Bi-LSTM model. Dependence plot showing the relation between **(B)** EVI in June and the corresponding SHAP value and **(C)** maximum temperature in April and its SHAP value.

The SHAP summary plot (**Figure 9A**) provides a dense summary of information from the SHAP analysis. The plot displayed the top 20 most influential features in order of importance from top to bottom, based on their overall impact on the model’s output. Each dot on the plot represents a single data point, with the dot’s color indicating the value of the feature—green for higher values and blue for lower values. The horizontal position of the dot represents the SHAP value of the corresponding feature for that data point. From the figure, it is evident that, besides EVI and precipitation accumulation during months before harvest, maximum temperature from March to July is also among the top 20 influencing features. This plot also visualizes how different feature values contribute to yield. For EVIs, data points with higher (green) values before harvest months had higher SHAP values, suggesting a positive correlation between SHAP and EVI values. On the other hand, lower precipitation and maximum temperatures (blue points in corresponding rows) during that period are generally associated with positive SHAP values, and higher precipitation and temperatures have negative SHAP values, indicating their negative co-relation.

The relation between features and yield value at a global scale can be further understood by a dependence plot, which is a scatter plot of the feature values and SHAP values for that feature. For instance, there is a positive linear relationship between EVI in June and the corresponding SHAP value (**Figure 9B**). This means the model tends to make higher predictions when EVI in June is higher, and the relation is consistent. Dependence plots can reveal nonlinear relationships between features and their impact on model outputs as well. For example, the dependence plot of maximum temperature in April (**Figure 9C**) indicates that maximum temperatures up to 21°C in April have a negligible impact on the model’s output. However, as temperatures rise beyond 21°C, the SHAP value for that feature decreases, suggesting that it pushes predictions towards lower values. While these relations were not evident from the summary plot, they are clear from the dependence.

**Figure 10:**
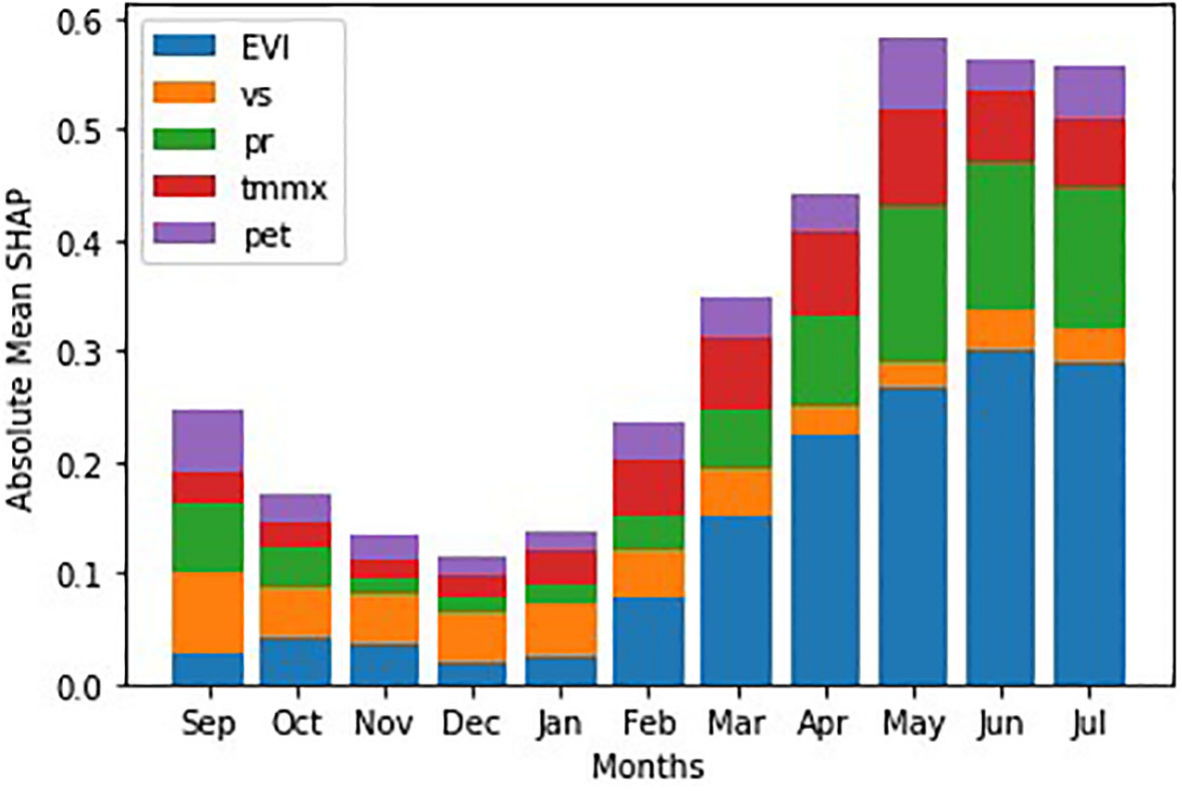
SHAP attribution over the winter wheat growing months.

We draw a stacked bar plot of absolute mean SHAP values to reveal how different features contribute to the model’s predictions over the months (**Figure 10**). Clearly, the overall impact of features is higher in months closer to harvest. Additionally, the plot also reveals which features were the most important in each month. For example, wind speed appears important in the initial months, but its impact diminishes as we approach harvest.

**Figure 11:**
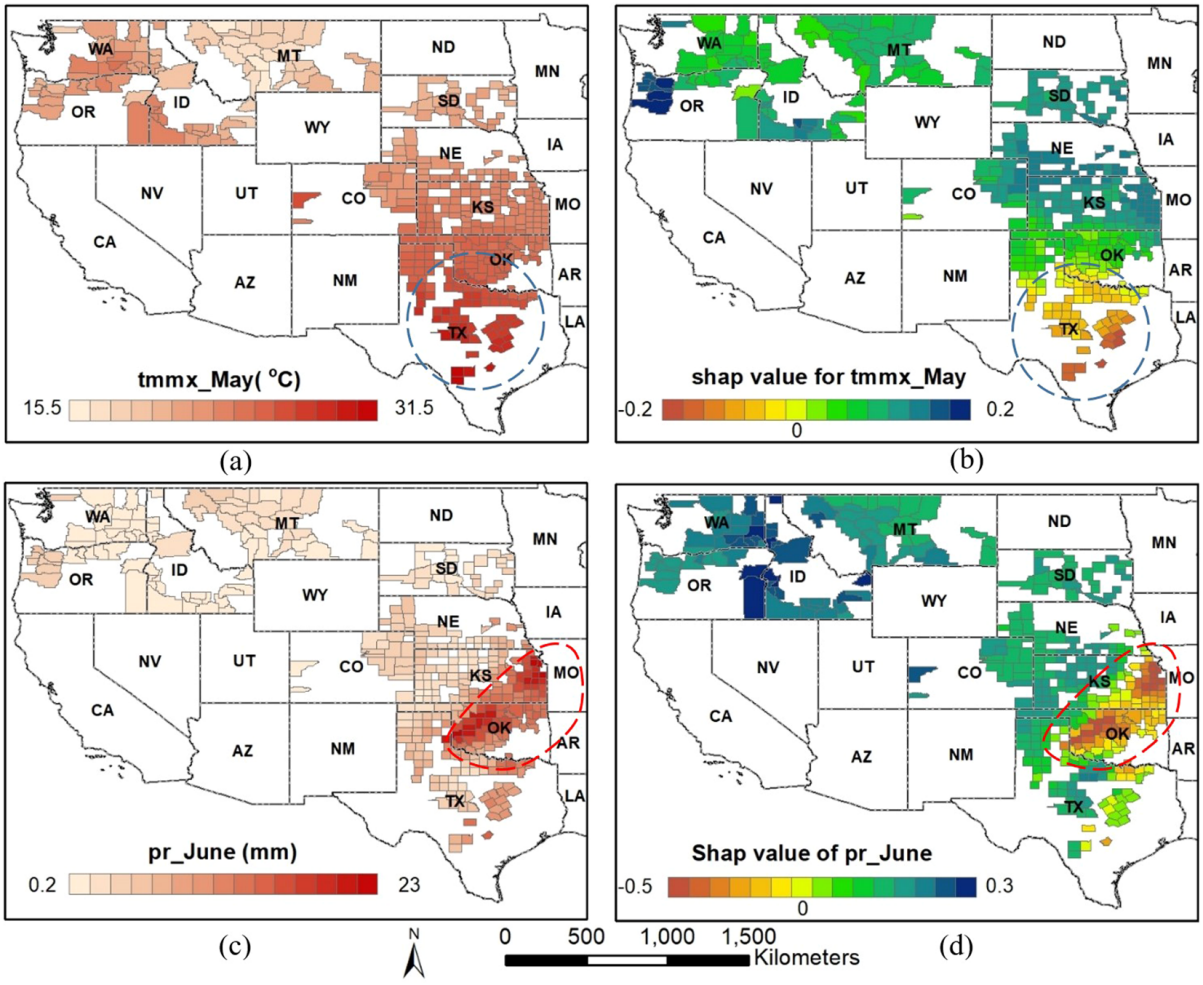
Spatial distribution of feature values **(A, C)** and corresponding SHAP values **(B, D)** for year 2021 for two selected features: maximum temperature in May and precipitation accumulation in June.

The map of feature values and corresponding SHAP values can be used to understand various key insights, including the spatial distribution of features and their impact on model output, as well as the relationship between them. Visual inspection also aids in identifying areas with distinct patterns, such as high positive or negative impacts by the feature. For instance, low SHAP values for maximum temperature in May in the area inside the blue box indicate a stronger negative impact on the model output within this region due to temperature (**Figure 11B**). Similarly, low SHAP values for rainfall in June in the area inside the red box suggest a greater negative impact on the model output in this area due to precipitation (**Figure 11D**). If we examine the corresponding feature values of that area, we can observe that the region inside the blue box (**Figure 11A**) has a relatively higher temperature, while the area inside the red box (**Figure 11C**) has relatively higher precipitation levels. The relation between these features and corresponding SHAP values aligns with the inverse relation depicted by the summary and dependence plot.

**Figure 12:**
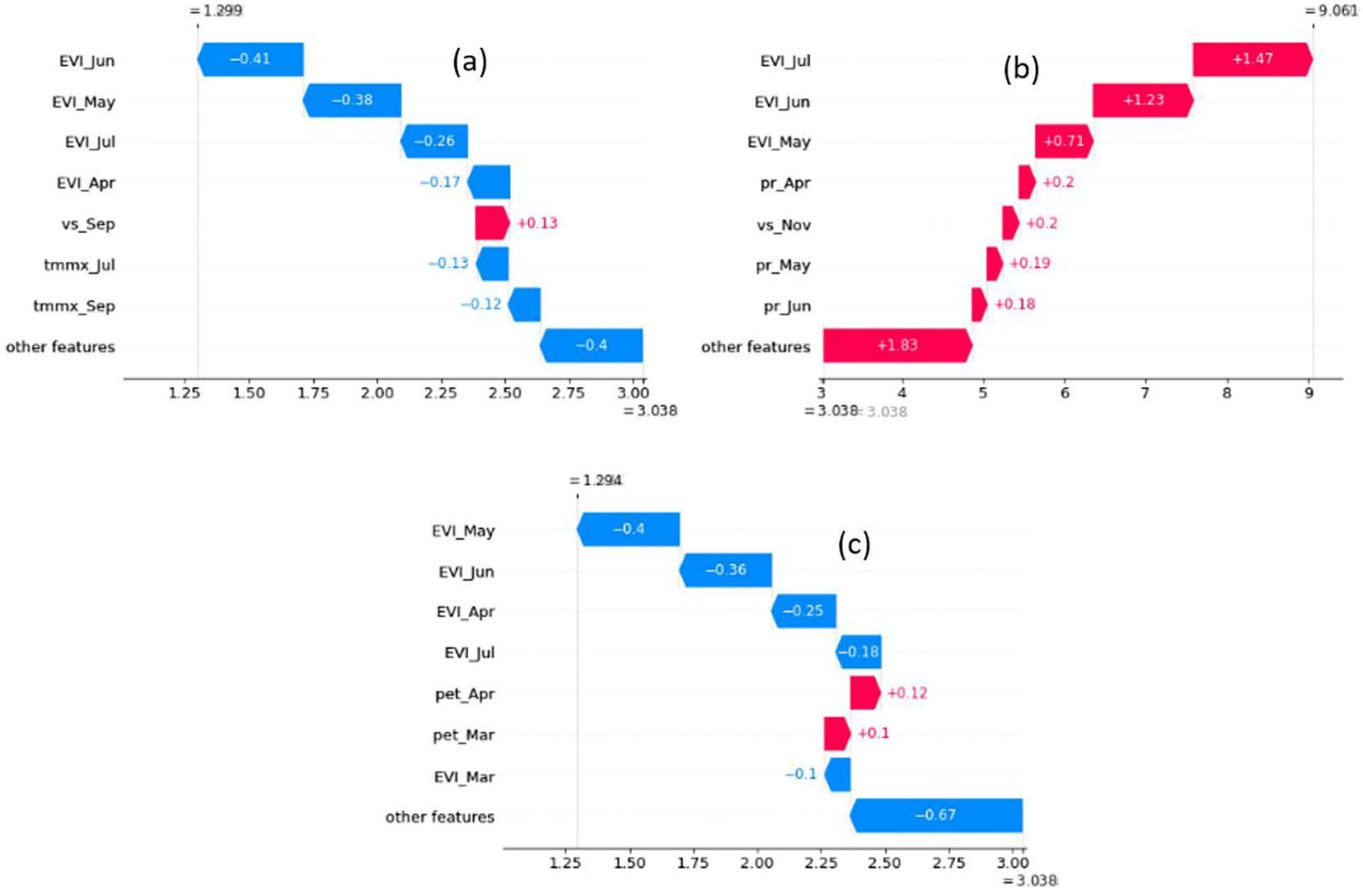
Waterfall plot is used to understand how the model made the prediction in three instances: **(A)** lowest yield prediction for 2020, **(B)** highest yield prediction for 2020, and **(C)** Relatively higher absolute error.

To explain individual predictions, we used waterfall plots, a built-in plot of the SHAP library. The base of each waterfall plot displays the expected value, which is the mean of all yield predictions. Subsequent rows illustrate the positive or negative contributions of the features, demonstrating how they shift the value from the mean model output to arrive at the specific prediction for that instance. We employed waterfall plots to examine three notable instances: the highest and lowest predictions for 2020, where the Bi-LSTM model exhibited its strongest performance, and a prediction characterized by a relatively higher absolute error (**Figure 12**).

The waterfall plot for the county with the highest yield prediction for 2020 (Twin Falls in Idaho) reveals positive SHAP values for all major features: EVI in May, June, and July, precipitation in April, May, and June, and windspeed in September. This suggests that all major determining climatic conditions were favorable for yield at that location and year. Additionally, the high EVI value also underscores the favorable conditions for a high yield. The waterfall plot for the lowest yield value in 2020 (Cottle in Texas) demonstrates that maximum temperatures in July and September were the primary environmental drivers responsible for the reduced yield. Notably, temperatures throughout the year at this location were higher than average, and dependence plots suggest a correlation between higher temperatures and lower yield predictions. The EVI values also captured unfavorable factors contributing to the decline in yield, as evidenced by negative SHAP values for EVI from May to July. Finally, the waterfall plot for the county with a relatively high error (Dawson in Texas) reveals that EVI values from March to July are the primary factors contributing to the lower-than-expected yield. This suggests that the EVI values for those months may not accurately reflect the actual yield at that location. The possible reasons could be the noise in the EVI data for that specific location or there is not sufficient training data to capture the specific variance in yield associated with those EVI values.

## 4 Discussion

The aim of the study was to develop and implement an explainable DL model capable of predicting crop yield with high accuracy and providing explanations for the predictions. To achieve this, we developed three state-of-the-art sequential DL models - LSTM, 1D CNN, and Bi-LSTM. We then employed three explainability techniques to gain insights into the model’s decision-making processes.

All the models employed in this study are powerful data-driven techniques and demonstrated strong performance in the winter wheat yield prediction (**Table 1**). RF is a traditional ensemble machine learning method, which generally requires feature engineering and cannot inherently learn sequential relationships. In contrast, the deep learning models—1D-CNN, LSTM, and Bi-LSTM—automatically learn features from raw data and effectively capture sequential dependencies between input features as well. The result showed that the Bi-LSTM model outperformed all other DL models and the RF model in terms of predictive performance and generalizability. The Bi-LSTM model achieved the highest R2 and the lowest MAE in all three test years (2019, 2020, and 2021) compared to the other models (**Table 1**). Visual analysis of scatter plots revealed underprediction in high-yield counties for all models and years. The underprediction of yields in high-yield counties has also been observed in other studies across crops, including corn (Ma et al., 2021), soybean (Maimaitijiang et al., 2020) and winter wheat (Wang et al., 2020)), and using various ML and DL methods. The reason for the underprediction could be attributed to an imbalanced training dataset with fewer high-yield samples. Notably, the Bi-LSTM model experienced the least underprediction in high-yield counties compared to other models. Further, the performance of all models declined for the year 2021. This is likely due to differences in data distribution for 2021 compared to the training years. For instance, the states of Idaho, Oregon, and Montana, which exhibited comparatively larger errors, experienced higher maximum temperatures during these three years than in other study years. Among the models, the Bi-LSTM demonstrated the smallest decline in performance, while the RF model struggled most to generalize effectively for the test year 2021. Additionally, for the Bi-LSTM model, the absolute error map shows the lowest error across the study area (**Figure 6**). This suggests that our Bi-LSTM model generalized well across a wide range of testing data and locations. The Bi-LSTM gain a deeper understanding of context by processing sequences from both ends and then combining them into a single enhanced representation (Dikshit et al., 2021; Graves and Schmidhuber, 2005). The promising performance and generalizability of the Bi-LSTM model in our study demands investigating into its potential for other crop types and study areas.

The explainability analysis revealed a range of significant findings, including insights into which features are given importance across various models employing the feature attribution method, the time when they are important, the nature of the relation between features and their impact (linear, nonlinear, positive, negative), and possible explanation of different instances such as high/low yield, high error in prediction. Comparison of LIME, SHAP, and IG-based attribution yielded consistent results. This agrees with the results of (Mateo-Sanchis et al., 2023), who similarly observed consistency between attribution provided by IG and SHAP for LSTM models in crop yield prediction. While LIME is model-agnostic, meaning it can be applied to various models, Integrated Gradients (IG) and DeepSHAP are specifically designed for neural networks. In terms of computational efficiency, IG was found to be the fastest, followed by LIME. SHAP was significantly more computationally intensive. Interestingly, the overall patterns of feature importance across DL models were also consistent. This contrasts with some earlier findings (Stenwig et al., 2022), who reported that different ML models considered different features to be the most important predictors of mortality in ICU. Al-Najjar et al. (2023) also reported the relationships between some features and their corresponding SHAP values were different for RF and SVM (positive correlation in one model and negative in another). The consistent attribution observed across our DL models is likely due to their shared ability to capture sequential patterns within the data and the fact that they were all trained on the same dataset. In contrast, the RF model exhibits different attribution patterns, prioritizing a few key variables while assigning lower importance to others. This difference in attribution patterns also supports the possibility that models designed to learn sequential relationships tend to assign similar levels of importance to features.

Regarding the importance of different features, across all three DL models, EVI measured between greenup and harvest (March to July) were the most influential factors for predicting yield. The RF model also indicated that EVI values from flowering to maturity (May and June) were among the top two most important features. The summary plot and dependence plot (**Figure 8**) were used to investigate the nature of the relation. There is a positive correlation between EVI and SHAP values, indicating that higher EVI values generally contribute to increased model predictions and vice versa. Particularly, EVI at June, which is the time of physiological maturity and has the highest contribution in the model, and this relationship is nearly linear. This aligns with previous findings, which show vegetation indices at the later stages of growth to be a crucial factor in determining yield and demonstrate a positive association (Joshi et al., 2023a, 2023c; Li et al., 2022). However, most of those studies relied on correlation analysis to identify the magnitude and the nature of the relationship. In contrast, our approach confirmed the relation from the trained ML models using an explainability approach.

Precipitation accumulation and maximum temperature during that period were the next most important features. Both features have a negative relationship with the model’s output. In fact, a primary environmental factor constraining wheat yield is the stress induced by high temperatures (Narayanan et al., 2016). Gibson and Paulsen (1999) reported a decrease in wheat yield of more than 10% per 1°C in mean temperature from 22.5 to 27.5°C. Interestingly, the dependence plot of maximum temperature in April (**Figure 8**) shows a nonlinear relationship with a negative slope with corresponding SHAP value. The plot shows that as temperatures rise beyond around 21°C, the SHAP value for that feature decreases, suggesting that as temperature increases beyond that point, the model predicts lower yield due to negative contribution from the temperature variable. The relation between precipitation and temperature and their impact on yield in our study area was further illustrated in **Figure 11**.

Our study demonstrates that XAI goes beyond merely providing global feature importance in crop yield prediction. We identified the contribution of features like EVI, temperature, and precipitation in predicting winter wheat yield and also explored the nature of the interaction. Further, the investigation of attribution values provided insight into how each model was making decisions and provided an explanation of different behaviors. For example, the RF model experienced a significant drop in accuracy in 2021 (**Table 1**). Examining the heatmap of feature importance revealed that the model focused on only a few features, neglecting valuable information in others (**Figure 8**). This could lead to the model being unable to capture diverse conditions and thus compromising its generalizability. XAI could potentially be used to improve the model as well. For example, our explainability analysis revealed that EVI from May to harvest strongly influences yield prediction, while EVI from November to January exhibits the least impact. This coincides with increased noise in vegetation index data during those winter months due to cloud cover and snow. A common approach to dealing with such a situation is to omit erroneous data, resulting in a decrease in data points for training and prediction. The findings suggest that we might use noisy data for those months or simply impute those values based on calculations from neighboring months, which would provide us with more data points for training and prediction without compromising the performance of the model. This requires further investigation. Furthermore, we used XAI to provide possible explanations for high-yield, low-yield, and erroneous yield prediction instances (**Figure 12**).

This study provides an approach to understanding the decision-making process of a DL yield prediction model. By offering transparency into the model’s predictions, it builds trust in the results. The approach also explored the complex interactions between different environmental and remote sensing variables determining yield. The outputs of explainability can be assessed against established domain knowledge to verify if the model is functioning as expected. If the insights contradict known knowledge, it may be necessary to re-evaluate the input features or the model architecture and training steps. This would lead more reliable yield predictions.

The strengths of this study include the use of different ML models and XAI techniques with in-depth analysis and visualization of attribution values to achieve high accuracy and understand how the model works as a whole and for individual predictions. The input features used in this study are globally available. This makes the approach adaptable to other crop types and regions, provided historical yield data is available for that region. To enhance the model’s performance and generalizability, incorporating additional features such as soil properties, management practices, and cultivar information can be beneficial. However, when integrating non-temporal data, directly applying sequential models may not be ideal. In such cases, alternative strategies, like employing hybrid models capable of learning from both sequential and non-sequential data, should be considered. The findings of the study must be interpreted considering a few limitations. The features used in this study were correlated, and the time-series data exhibited autocorrelation. Although the prediction models used are capable of handling such complex datasets, the attribution value of explainability methods might have been influenced by this condition. A further limitation of the explainability methods is that they do not inherently capture the internal workings of a model, and the identified relationships between features and predictions are not necessarily causal. Features excluded from the model that influence both included features and the outcome can lead to misinterpretations of these relationships. Moreover, attribution values show how features contribute to model predictions, but if the model does not have satisfactory accuracy for some instances, the explanation provided by them might not accurately reflect the contributions of input features to the target variable.

## 5 Conclusion

Accurate and transparent crop yield prediction is important to address global food security concerns. In this study, we employed the XAI approach to achieve accurate and explainable winter wheat yield prediction. Our Bi-LSTM model outperformed LSTM, 1D-CNN, and RF models in terms of predictive performance as measured by R2 and MAE, and demonstrated superior generalizability across various locations and yield ranges. Explainability analysis revealed that all sequential DL models learn similar features, as indicated by different attribution methods. The study identified the importance of EVI, temperature, and precipitation during the later stages of crop growth in determining winter wheat yield. EVI during this period exhibited a strong positive correlation with wheat yield, while temperature and precipitation during the same period showed a negative correlation. These findings align with those of previous studies and expert opinions. Furthermore, we demonstrated that XAI methods can be used to understand instances such as high- and low-yield samples, provide possible explanations for erroneous predictions, and identify regions impacted by specific stresses. The main contribution of this study is the development and implementation of an explainable Bi-LSTM model for crop yield prediction at a regional scale, providing both high accuracy and intuitive explanations of the predictions. This combination of high accuracy and interpretability builds trust in the yield prediction made by the models and providing valuable information for farmers, businesses and policymakers. The explainability approach presented in this study can be applied to any yield prediction DL models to improve their transparency and reliability. Future study should investigate the application of the Bi-LSTM model across different regions and crop types and expand XAI to focus on causal relationships, limiting the effects of collinear features.

## Data Availability

The original contributions presented in the study are included in the article/supplementary material. Further inquiries can be directed to the corresponding authors.

## References

[B1] Abadi M. Barham P. Chen J. Chen Z. Davis A. Dean J.. (2016). “Tensorflow: A system for large-scale machine learning,” in 12th {USENIX} symposium on operating systems design and implementation ({OSDI} 16), Savannah, GA, USA: Conference Proceedings. November 2–4, 2016.

[B2] Abatzoglou J. T. Dobrowski S. Z. Parks S. A. Hegewisch K. C. (2018). TerraClimate, a high-resolution global dataset of monthly climate and climatic water balance from 1958–2015. Sci. Data 5, 1–12. doi: 10.1038/sdata.2017.191 29313841 PMC5759372

[B3] Al-Najjar H. A. Pradhan B. Beydoun G. Sarkar R. Park H.-J. Alamri A. (2023). A novel method using explainable artificial intelligence (XAI)-based Shapley Additive Explanations for spatial landslide prediction using Time-Series SAR dataset. Gondwana Res. 123, 107–124. doi: 10.1016/j.gr.2022.08.004

[B4] Baehrens D. Schroeter T. Harmeling S. Kawanabe M. Hansen K. Müller K.-R. (2010). How to explain individual classification decisions. J. Mach. Learn. Res. 11, 1803–1831.

[B5] Beck H. E. Zimmermann N. E. McVicar T. R. Vergopolan N. Berg A. Wood E. F. (2018). Present and future Köppen-Geiger climate classification maps at 1-km resolution. Scientific data 5 (1), 1–12. 30375988 10.1038/sdata.2018.214 PMC6207062

[B6] Bengio Y. Grandvalet Y. (2003). No unbiased estimator of the variance of k-fold cross-validation. Advances in Neural Information Processing Systems, 16.

[B7] Breiman L. (2001). Random forests. Mach. Learn. 45, 5–32. doi: 10.1023/A:1010933404324

[B8] Chen B. Zheng H. Wang L. Hellwich O. Chen C. Yang L.. (2022). A joint learning Im-BiLSTM model for incomplete time-series Sentinel-2A data imputation and crop classification. Int. J. Appl. Earth Observation Geoinformation 108, 102762. doi: 10.1016/j.jag.2022.102762

[B9] Corbeels M. Chirat G. Messad S. Thierfelder C. (2016). Performance and sensitivity of the DSSAT crop growth model in simulating maize yield under conservation agriculture. Eur. J. Agron. 76, 41–53. doi: 10.1016/j.eja.2016.02.001

[B10] Dikshit A. Pradhan B. Alamri A. M. (2021). Pathways and challenges of the application of artificial intelligence to geohazards modelling. Gondwana Res. 100, 290–301. doi: 10.1016/j.gr.2020.08.007

[B11] FAO. (2022). Staple foods: What do people eat. Available online at: https://www.fao.org/3/u8480e/U8480E07.HTM (Accessed November 23, 2023).

[B12] FAO, S. D. (2021). World Food and Agriculture Statistical Yearbook. Food and Agriculture Organization of the United Nations.

[B13] Fel T. Hervier L. Vigouroux D. Poche A. Plakoo J. Cadene R.. (2022). Xplique: A deep learning explainability toolbox. arXiv preprint arXiv:2206.04394.

[B14] Filho H. C. C. Júnior O. A. C. de Carvalho O. L. F. de Bem P. P. de Moura R. S. de Albuquerque A. O.. (2020). Rice crop detection using LSTM, Bi-LSTM, and machine learning models from Sentinel-1 time series. Remote Sens. 12. doi: 10.3390/RS12162655

[B15] Folberth C. Skalský R. Moltchanova E. Balkovič J. Azevedo L. B. Obersteiner M.. (2016). Uncertainty in soil data can outweigh climate impact signals in global crop yield simulations. Nat. Commun. 7, 11872. doi: 10.1038/ncomms11872 27323866 PMC4919520

[B16] Gibson L. Paulsen G. (1999). Yield components of wheat grown under high temperature stress during reproductive growth. Crop Sci. 39, 1841–1846. doi: 10.2135/cropsci1999.3961841x

[B17] Goodfellow I. Bengio Y. Courville A. (2016). Deep learning (London, England: MIT press).

[B18] Gorelick N. Hancher M. Dixon M. Ilyushchenko S. Thau D. Moore R. (2017). Google Earth Engine: Planetary-scale geospatial analysis for everyone. Remote Sens. Environ. 202, 18–27. doi: 10.1016/j.rse.2017.06.031

[B19] Graves A. Schmidhuber J. (2005). Framewise phoneme classification with bidirectional LSTM and other neural network architectures. Neural Networks 18, 602–610. doi: 10.1016/j.neunet.2005.06.042 16112549

[B20] Guerif M. Philipe O. Delécolle R. (1985). “Statistical modelling of winter yield at a regional scale,” in Wheat Growth and Modelling. Eds. Day W. Atkin R. K. (Springer, US), 371–379. doi: 10.1007/978-1-4899-3665-3_33

[B21] Hochreiter S. Schmidhuber J. (1997). Long short-term memory. Neural Comput. 9, 1735–1780. doi: 10.1162/neco.1997.9.8.1735 9377276

[B22] Hoffman L. A. Etienne X. L. Irwin S. H. Colino E. V. Toasa J. I. (2015). Forecast performance of WASDE price projections for US corn. Agric. Economics 46, 157–171. doi: 10.1111/agec.2015.46.issue-S1

[B23] Huang J. Tian L. Liang S. Ma H. Becker-Reshef I. Huang Y.. (2015). Improving winter wheat yield estimation by assimilation of the leaf area index from Landsat TM and MODIS data into the WOFOST model. Agric. For. Meteorology 204, 106–121. doi: 10.1016/j.agrformet.2015.02.001

[B24] Isengildina-Massa O. Irwin S. H. Good D. L. Gomez J. K. (2008). The impact of situation and outlook information in corn and soybean futures markets: Evidence from WASDE reports. J. Agric. Appl. Economics 40, 89–103.

[B25] Jiang H. Hu H. Zhong R. Xu J. Xu J. Huang J.. (2020). A deep learning approach to conflating heterogeneous geospatial data for corn yield estimation: A case study of the US Corn Belt at the county level. Global Change Biol. 26, 1754–1766. doi: 10.1111/gcb.14885 31789455

[B26] Joshi D. R. Clay S. A. Sharma P. Rekabdarkolaee H. M. Kharel T. Rizzo D. M.. (2023c). Artificial intelligence and satellite-based remote sensing can be used to predict soybean (Glycine max) yield. Agron. J.

[B27] Joshi A. Pradhan B. Chakraborty S. Behera M. D. (2023a). Winter wheat yield prediction in the conterminous United States using solar-induced chlorophyll fluorescence data and XGBoost and random forest algorithm. Ecol. Inf. 77, 102194. doi: 10.1016/j.ecoinf.2023.102194

[B28] Joshi A. Pradhan B. Gite S. Chakraborty S. (2023b). Remote-sensing data and deep-learning techniques in crop mapping and yield prediction: A systematic review. Remote Sens. 15, 2014. doi: 10.3390/rs15082014

[B29] Keating B. A. Carberry P. S. Hammer G. L. Probert M. E. Robertson M. J. Holzworth D.. (2003). An overview of APSIM, a model designed for farming systems simulation. Eur. J. Of Agron. 18, 267–288. doi: 10.1016/S1161-0301(02)00108-9

[B30] Khanal S. Fulton J. Klopfenstein A. Douridas N. Shearer S. (2018). Integration of high resolution remotely sensed data and machine learning techniques for spatial prediction of soil properties and corn yield. Comput. Electron. Agric. 153, 213–225. doi: 10.1016/j.compag.2018.07.016

[B31] Kohavi R. (1995). A study of cross-validation and bootstrap for accuracy estimation and model selection. Morgan Kaufman Publishing.

[B32] Kushal K. Khanal S. (2023). Agricultural productivity and water quality tradeoffs of winter cover crops at a landscape scale through the lens of remote sensing. J. Environ. Manage. 330, 117212. doi: 10.1016/j.jenvman.2022.117212

[B33] Kuwata K. Shibasaki R. (2016). Estimating corn yield in the United States with MODIS EVI and machine learning methods. ISPRS Ann Photogrammetry Remote Sens. Spatial Information Sci. III-8, 131–136. doi: 10.5194/isprsannals-III-8-131-2016

[B34] Lapuschkin S. Wäldchen S. Binder A. Montavon G. Samek W. Müller K.-R. (2019). Unmasking Clever Hans predictors and assessing what machines really learn. Nat. Commun. 10, 1096. doi: 10.1038/s41467-019-08987-4 30858366 PMC6411769

[B35] Li Z. Ding L. Xu D. (2022). Exploring the potential role of environmental and multi-source satellite data in crop yield prediction across Northeast China. Sci. Total Environ. 815, 152880. doi: 10.1016/j.scitotenv.2021.152880 34998760

[B36] Lobell D. B. Thau D. Seifert C. Engle E. Little B. (2015). A scalable satellite-based crop yield mapper. Remote Sens. Environ. 164, 324–333. doi: 10.1016/j.rse.2015.04.021

[B37] Lundberg S. M. Lee S.-I. (2017). A unified approach to interpreting model predictions. Adv. Neural Inf. Process. Syst. 30.

[B38] Ma Y. Zhang Z. Kang Y. Özdoğan M. (2021). Corn yield prediction and uncertainty analysis based on remotely sensed variables using a Bayesian neural network approach. Remote Sens. Environ. 259, 112408. doi: 10.1016/j.rse.2021.112408

[B39] Ma J. Ding Y. Cheng J. C. Jiang F. Tan Y. Gan V. J.. (2020). Identification of high impact factors of air quality on a national scale using big data and machine learning techniques. Journal of Cleaner Production 244, 118955.

[B40] Maimaitijiang M. Sagan V. Sidike P. Hartling S. Esposito F. Fritschi F. B. (2020). Soybean yield prediction from UAV using multimodal data fusion and deep learning. Remote Sens. Environ. 237, 111599. doi: 10.1016/j.rse.2019.111599

[B41] Mateo-Sanchis A. Adsuara J. E. Piles M. Munoz-Marí J. Perez-Suay A. Camps-Valls G. (2023). Interpretable long short-term memory networks for crop yield estimation. IEEE Geosci. Remote Sens. Lett. 20, 1–5. doi: 10.1109/LGRS.2023.3244064

[B42] Matin S. S. Pradhan B. (2021). Earthquake-induced building-damage mapping using Explainable AI (XAI). Sensors 21, 4489. doi: 10.3390/s21134489 34209169 PMC8271973

[B43] Muruganantham P. Wibowo S. Grandhi S. Samrat N. H. Islam N. (2022). A systematic literature review on crop yield prediction with deep learning and remote sensing. Remote Sens. 14, 1990. doi: 10.3390/rs14091990

[B44] Narayanan S. Tamura P. J. Roth M. R. Prasad P. V. Welti R. (2016). Wheat leaf lipids during heat stress: I. High day and night temperatures result in major lipid alterations. Plant Cell Environ. 39, 787–803. doi: 10.1111/pce.12649 26436679 PMC5102054

[B45] NASS. (2022). USDA National Agricultural Statistics Service Cropland Data Layer. Available online at: https://nassgeodata.gmu.edu/CropScape (Accessed November 23, 2023).

[B46] Pedregosa F. Varoquaux G. Gramfort A. Michel V. Thirion B. Grisel O.. (2011). Scikit-learn: machine learning in python. J. Mach. Learn. Res. 12, 2825–2830.

[B47] Ramos A. P. M. Osco L. P. Furuya D. E. G. Gonçalves W. N. Santana D. C. Teodoro L. P. R.. (2020). A random forest ranking approach to predict yield in maize with uav-based vegetation spectral indices. Comput. Electron. Agric. 178, 105791. doi: 10.1016/j.compag.2020.105791

[B48] Ribeiro M. T. Singh S. Guestrin C. (2016). “Why should i trust you?” Explaining the predictions of any classifier,” in Proceedings of the 22nd ACM SIGKDD international conference on knowledge discovery and data mining, San Francisco California USA: Conference Proceedings. August 13 - 17, 2016.

[B49] Saini P. Nagpal B. Garg P. Kumar S. (2023). CNN-BI-LSTM-CYP: A deep learning approach for sugarcane yield prediction. Sustain. Energy Technol. Assessments 57, 103263. doi: 10.1016/j.seta.2023.103263

[B50] Schuster M. Paliwal K. K. (1997). Bidirectional recurrent neural networks. IEEE Trans. Signal Process. 45, 2673–2681. doi: 10.1109/78.650093

[B51] Schwalbert R. A. Amado T. Corassa G. Pott L. P. Prasad P. V. V. Ciampitti I. A. (2020). Satellite-based soybean yield forecast: Integrating machine learning and weather data for improving crop yield prediction in southern Brazil. Agric. For. Meteorology 284, 107886. doi: 10.1016/j.agrformet.2019.107886

[B52] Shapley L. (1953). Quota solutions op n-person games1, Vol. 343. Eds. Artin E. Morse M. (St Monica, Caifornia USA: The Rand Corporation).

[B53] Sherrick B. J. Lanoue C. A. Woodard J. Schnitkey G. D. Paulson N. D. (2014). Crop yield distributions: fit, efficiency, and performance. Agric. Finance Rev. 74.

[B54] Simonyan K. Vedaldi A. Zisserman A. (2013). Deep inside convolutional networks: Visualizing image classification models and saliency maps. arXiv preprint arXiv:1312.6034.

[B55] Srivastava A. K. Safaei N. Khaki S. Lopez G. Zeng W. Ewert F.. (2022). Winter wheat yield prediction using convolutional neural networks from environmental and phenological data. Sci. Rep. 12, 3215. doi: 10.1038/s41598-022-06249-w 35217689 PMC8881605

[B56] Stenwig E. Salvi G. Rossi P. S. Skjærvold N. K. (2022). Comparative analysis of explainable machine learning prediction models for hospital mortality. BMC Med. Res. Method. 22, 1–14. doi: 10.1186/s12874-022-01540-w PMC8882271 35220950

[B57] Sundararajan M. Taly A. Yan Q. (2017). “Axiomatic attribution for deep networks,” in International conference on machine learning, Sydney AUSTRALIA: Conference Proceedings. Aug 6 - 11, 2017.

[B58] USDA. (2023). United States Department of Agriculture National Agricultural Statistics Service. Available at: https://quickstats.nass.usda.gov (Accessed November 23, 2023).

[B59] USDA. (2021). U.S. Wheat Exports in 2021. Available online at: https://www.fas.usda.gov/commodities/wheat (Accessed November 11, 2023).

[B60] USDA-NASS. (2021). Crop Production. Available online at: https://downloads.usda.library.cornell.edu/usda-esmis/files/tm70mv177/qf85pc34p/vh53xv71m/crop1021.pdf (Accessed October 2021).

[B61] Wang A. X. Tran C. Desai N. Lobell D. Ermon S. (2018). “Deep transfer learning for crop yield prediction with remote sensing data,” in 1st ACM SIGCAS Conference on Computing and Sustainable Societies, COMPASS 2018, San Jose CA USA: Conference Proceedings. June 20–22, 2018.

[B62] Wang Y. Zhang Z. Feng L. Du Q. Runge T. (2020). Combining multi-source data and machine learning approaches to predict winter wheat yield in the conterminous United States. Remote Sens. 12, 1232. doi: 10.3390/RS12081232

[B63] Wolanin A. Mateo-Garciá G. Camps-Valls G. Gómez-Chova L. Meroni M. Duveiller G.. (2020). Estimating and understanding crop yields with explainable deep learning in the Indian Wheat Belt. Environ. Res. Lett. 15, 024019. doi: 10.1088/1748-9326/ab68ac

